# *Still Heart* Encodes a Structural HMT, SMYD1b, with Chaperone-Like Function during Fast Muscle Sarcomere Assembly

**DOI:** 10.1371/journal.pone.0142528

**Published:** 2015-11-06

**Authors:** Kendal Prill, Pamela Windsor Reid, Serene L. Wohlgemuth, David B. Pilgrim

**Affiliations:** Department of Biological Sciences, University of Alberta, Edmonton, Alberta, Canada; Washington State University, UNITED STATES

## Abstract

The vertebrate sarcomere is a complex and highly organized contractile structure whose assembly and function requires the coordination of hundreds of proteins. Proteins require proper folding and incorporation into the sarcomere by assembly factors, and they must also be maintained and replaced due to the constant physical stress of muscle contraction. Zebrafish mutants affecting muscle assembly and maintenance have proven to be an ideal tool for identification and analysis of factors necessary for these processes. The *still heart* mutant was identified due to motility defects and a nonfunctional heart. The cognate gene for the mutant was shown to be *smyd1b* and the *still heart* mutation results in an early nonsense codon. SMYD1 mutants show a lack of heart looping and chamber definition due to a lack of expression of heart morphogenesis factors *gata4*, *gata5* and *hand2*. On a cellular level, fast muscle fibers in homozygous mutants do not form mature sarcomeres due to the lack of fast muscle myosin incorporation by SMYD1b when sarcomeres are first being assembled (19hpf), supporting SMYD1b as an assembly protein during sarcomere formation.

## Introduction

The sarcomere is a complex and highly organized structure where distinct protein filaments rely on the involvement and coordination of hundreds of other proteins for proper patterning, assembly and maintenance. Sarcomere proteins fall into three broad categories including contractile proteins, such as actin and myosin; structural attachment and anchoring components, such as integrins, α-actinin and myomesin, and patterning and folding chaperones such as Hsp90 [1–3]. While the function and activity of these proteins is well established in the mature sarcomere, the mechanisms by which the sarcomere proteins are assembled and initially patterned as the myocyte differentiates are still a matter of conjecture.

Many human myopathies are due to muscle weakness or progressive degeneration of the muscle due to the inability to maintain or replace sarcomere proteins [4, 5]. The sarcomere is a continuously active structure with high levels of protein turnover. When a structural sarcomeric protein begins losing integrity, a newly formed and folded protein replaces it; a process facilitated by sarcomere chaperones and their cofactors [6]. Without chaperones for maintenance of sarcomere proteins, this complex contractile structure would denature and muscle function would cease [7–10]. Some chaperones are required for assembly of the sarcomere, others for maintenance (SMYD2) and some have been proposed to perform both functions throughout muscle development (UNC45b and HSP90a1)[6, 11–13], however many factors remain unidentified [14]. Of those assembly and maintenance factors that have been isolated, molecular function and clients of these proteins often remain unclear.

A powerful approach to understanding this patterning process has been to knock down known or suspected factors in cell culture or animal models and determine the earliest detectable defect, but this can only test components identified by criteria such as gene expression or biochemical purification to be enriched in the developing sarcomere. Embryonic lethality of mammalian embryos bearing null mutations in many sarcomeric proteins hampers gene discovery screens in mice. The zebrafish provides a powerful platform for such forward screens as the embryo is able to survive for several days without cardiac or trunk muscle function. An extensive genetic analysis of developmental mutants published in 1996 identified hundreds of mutations affecting locomotive behavior [14]. In the majority of mutants, defects are seen either in trunk muscle, as seen in motility mutants *sloth*, *twitch once* and *techno trousers*, or cardiac muscle in mutants *valentine*, *weak atrium* and *beach bum* [14, 15]. However, a small subset (less than 15%) of motility mutants have defects in both heart and trunk muscle [14], suggesting that there may be only a small number of shared factors that work in both tissues. These dual cardiac and skeletal mutants suggest a model where one pathway responsible for patterning or sarcomere assembly is shared between both muscle types early muscle development.

We have been particularly interested in those non-myofilament proteins, such as myosin-specific chaperone UNC-45, that function early in the patterning of striated muscle, as they are likely candidates for factors necessary for establishing the patterning, alignment and register of the early myofibrils [10, 16–19]. Those zebrafish mutants affecting both cardiac and skeletal muscle, and whose myofibers show early disorganization without obvious defects to somite morphogenesis would be excellent candidates for factors in a rudimentary chaperone-dependent assembly process common to all sarcomeres, or alternatively to a common mechanism for sarcomere homeostasis.

The SMYD family of proteins has been recently identified as contributing to sarcomere assembly and maintenance [20, 21]. While the presence of MYND and SET domains in the SMYD protein suggests a role as a histone methyltransferase (HMT) [22, 23], recent work has demonstrated that there are other protein binding domains within this family that are consistent with chaperone-like behavior [20, 21, 24]. SMYD1 is expressed in the heart and skeletal muscle in both mouse and zebrafish; knockdown in mice leads to a fatal cardiomyopathy while a knockdown of *smyd1b* in zebrafish leads to a dual-skeletal and cardiomyopathy that is lethal at 7 days past fertilization [22, 25]. Zebrafish SMYD1 mutants share similar phenotypes to other muscle chaperone mutants, *steif* and *sloth*, that have mutations in *unc45b* and *hsp90a1*, respectively [2, 10, 13]. Mutations in either of these assembly/maintenance proteins results in paralysis (*sloth/hsp90a1*) or a dual myopathy of both cardiac and skeletal tissues (*steif/unc45b*) [2, 10, 13].

One of the mutants identified in the 1996 screen, *still heart* (*sth*) [14] had characteristics (a nonfunctional heart and reduced motility) similar to existing mutants known to affect early sarcomere patterning and assembly (*steif*/*unc45b* and *sloth*/*hsp90a1*) [2, 10]. Here we show that fast muscle fibers in *sth* embryos do not form mature sarcomeres as a result of the lack of fast muscle myosin. Additionally, fast myosin is never incorporated into the early premyofibers when sarcomeres are first being assembled (19hpf). Genetic mapping and complementation of the *sth* mutant shows that the phenotype is due to a nonsense mutation in the *smyd1b* gene. The expression of muscle chaperones *unc45b* and *hsp90a1* do not increase until 24hpf, suggesting that these proteins do not increase their expression in response to an absence of SMYD1b (at 19hpf) but are rather responding to increased levels of misfolded myosins[26–28]. This data, along with existing expression data from other systems, suggests that SMYD1b has the role of a thick-filament assembly protein during sarcomere formation.

## Materials and Methods

### Ethics Statement

All protocols were performed according to the guidelines stated by the Canadian Council for Animal Care and the protocols approved by the Animal Care and Use Committee of the University of Alberta (Fish Research License: 14–0101 FR).

### Zebrafish Line Maintenance

The Nuüsslein-Volhard lab identified the *still heart* ^*tm123a*^(*sth*) zebrafish mutant in the Tubingen (TB) genetic background from an ENU mutagenesis screen [14]. The *sth* ^*tm123a*^ strain was crossed to the wild-type AB strain in our lab and further bred to the WIK (Wild Indian Karyotype) strain for microsatellite mapping [29, 30]. The *flatline* ^*zf340*^ mutant strain, with a T to A transversion mutant in exon 2, was generated from a ENU mutagenesis screen [14] and studied/maintained by the Rottbauer lab at the University of Ulm, Germany [20]. This mutant strain was a generous gift from Dr. Wolfgang Rottbauer. The *still heart* strain was maintained as adult heterozygotes in a recycling water aquatic facility and bred and maintained according to standard protocols [31]. Embryos were collected and maintained at 28.5°C in standard Zebrafish Embryo Medium for up to 7 days before collected for experiment preparation.

### Complementation Crossing

Adult heterozygous *still heart* and adult heterozygous *flatline* mutants were crossed in three separate pairings (1 heterozygous male *still heart* to 1 heterozygous female *flatline*) and the fertilized embryos were collected for phenotype scoring. Crosses between *still heart* and *flatline* heterozygotes gave broods with wild type and mutant phenotypes, consistent with Mendelian ratios.

### Touch Test

Adult heterozygous still heart mutants were crossed and fertilized embryos were collected for scoring. Embryos were observed for tail movements (flicking of the tail or rolling over), dechorionated and touched with forceps to induce movement. Embryos that showed no signs of tail movements on their own or by being touched with forceps were separated onto a plate designated “mutant”. Embryos that did move on their own or by touch stimulation were separated onto a plate designated “wt”. Scoring was done between 19 and 22 hpf to measure skeletal muscle integrity independently from heart function (heart does not start beating until 24 hpf). Scored embryos were maintained at 28.5°C in standard Zebrafish Embryo Medium until 24 and 48 hpf for identification of mutants and wild type siblings.

### Mutant Sequencing

Homozygous *still heart* mutants and their siblings were collected at 2 dpf, after the phenotype becomes apparent, for RNA extraction using TRIzol (Life Technologies, Burlington, ON, Canada). *smyd1b* (GenBank:CT027631) cDNA was sequenced from homozygous mutant and wild-type sibling RNA extractions that were reverse transcribed. The mutation present in cDNA was confirmed by genomic sequencing of an exon 1 RT/PCR product in homozygous *sth* mutants and their wild type siblings. Genomic sequencing was performed 10 times from 10 individual embryos. cDNA sequencing was performed 10 times from RNA extractions of 30 embryos. All sequencing was performed at the Molecular Biology Service Unit at the University of Alberta on a Sanger DNA Sequencer (ABI 3730). Primer sequences are available in S1 Table.

### In situ hybridization and immunostaining

*In situ* hybridization was performed on whole-mount zebrafish embryos fixed overnight in 4% paraformaldehyde (PFA), as previously published [32], using antisense RNA probes synthesized by in vitro transcription using SP6, T7 or T3 RNA polymerase. Probes used were zebrafish *unc45b* [13] (GenBank:CR847826), *hsp90a1* [33] (GenBank:BC075757), *smyhc1* [34] (GenBank:CU929259) and *myhc4* [35] (GenBank:BX511237). For immunostaining, embryos were staged morphologically, fixed for 1 hour at room temperature in 4% PFA. Immunostaining was carried out as previously described [33] with antibodies against fast-muscle myosin (1/10 F310, DSHB, Iowa, USA), slow-muscle myosin (1/10 F59, DSHB, Iowa, USA). All experiments were carried out 3 separate times, from 3 individual broods with a minimum of 15 embryos per condition (total = 45 wt and 45 *sth* embryos per probe/antibody). Primer sequences are available in S1 Table.

### RNA extractions and qPCR

RNA extractions were performed on collections of a minimum of 30 embryos that were flash frozen in liquid nitrogen before using a TRIzol RNA extraction. RNA was standardized to one concentration for all samples before proceeding to cDNA synthesis using a qScript cDNA synthesis kit (Quanta Biosciences, Gaithersberg, MD, USA). cDNA was diluted to 5ng/ul for Quantitative PCR. *ef1a* was used as the endogenous control for normalization of samples and expression of genes of interest. qPCR and data analysis was carried out using the Illumina Eco Real-Time PCR system. Three replicates of expression for each gene were generated for each N number of WT and *still heart* embryo collections. Error bars represent the standard deviation of the WT and *still heart* gene expression replicates generated from each qPCR. Primer sequences are available in S1 Table.

*Raw data can be found at* http://dx.doi.org/10.6084/m9.figshare.1454588

## Results

### *Still heart* has defects in both heart and skeletal muscle structure and function as a result of a mutation in *smyd1b*

**Fig 1:**
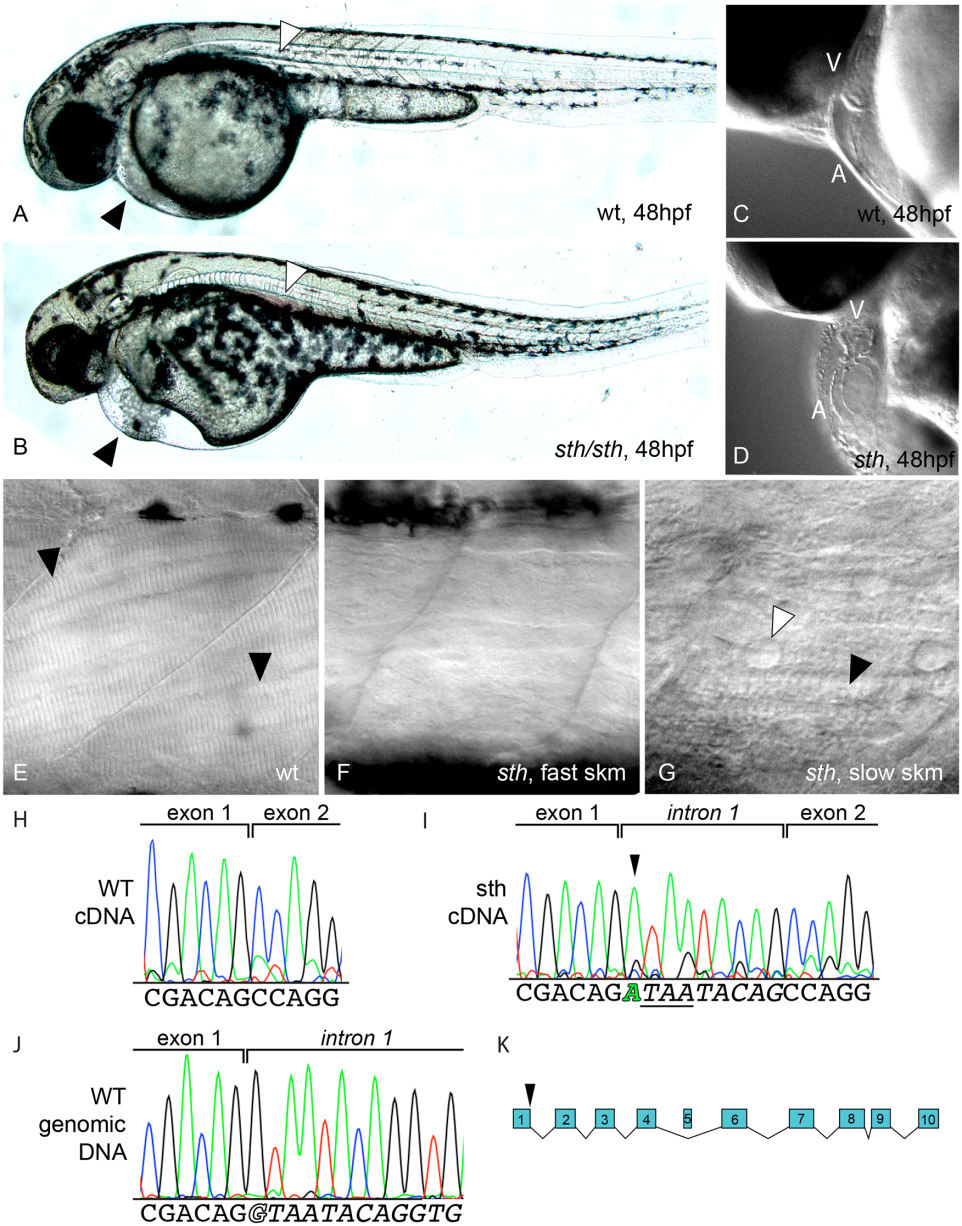
*Still heart*, a *smyd1b* mutant, has defects in heart and fast skeletal muscle tissue. A lateral view of 48hpf wild type (A) and *still heart* (*sth*) mutants (B), which have pericardial edema, small eyes, malformed head and reduced motility. Black arrowheads highlight the pericardial edema in sth mutants and the absence of edema in wild type. White arrowheads indicate blood pooling in the mutant and the absence of pooling in wild type. (C&D) *Sth* mutant hearts are underdeveloped and do not beat. (E-G) Examination of lateral myofibers at 5dpf under DIC microscopy revealed striations, indicative of fully formed sarcomeres, are visible in the myofibers of wild type muscle (E, black arrowheads), while absent in the fast muscle of *still heart* mutants (F); striations are present in *sth* slow muscle (G, black arrowhead) but are disturbed by nuclei and fluid-filled spaces (G, white arrowhead). Sequencing of *smyd1b* cDNA from wild type embryos (H) and *sth* mutant embryos (I) revealed a 9 nucleotide insertion between exon 1 and 2 in the *smyd1b* mRNA, creating an in-frame stop codon (I, underlined sequence). The insertion is the first 9 nucleotides of intron one as sequenced from wild type smyd1b genomic sequence (J). This is a result of a transition mutation in the splice donor site of intron 1 (I, green letter in sequence, J, outlined letter in sequence). This results in a premature truncation of the SMYD1b protein after exon 1 (K).

To identify and understand genes involved in early muscle developmental that may be shared between cardiac and skeletal muscle, we re-examined a zebrafish mutant, *still heart (sth)*, identified in a large-scale phenotypic screen [14]. *Still heart* mutants have a nonfunctional heart, pericardial edema, small eyes, malformed head and reduced motility (phenotype is lethal at 1 week) (Fig 1A and 1B). The *sth* phenotype is progressive and the condition of this mutant worsens over time as the pericardial edema spreads to the yolk, the head becomes further deformed and the frequency of a motility response to being touched decreases. Homozygous mutants, at 48 hpf (hours post fertilization), do not swim away after being touched but are only capable of a vibrational response. A heartbeat is never seen during development but two chambers do form although smaller than the wild type (Fig 1C and 1D). The progressive effect of the mutant is also visible in skeletal muscle tissue. At 24hpf, *sth* fast muscle tissue lacks striation in myofibrils, which suggests a defect in sarcomere assembly; slow-twitch myofibrils exhibit striation and appear normal. By 5 dpf, fast-twitch myofibrils are disorganized and both fast and slow muscle tissue is riddled with fluid-filled vacuoles (Fig 1E–1G). Part of the progressive nature of the *sth* phenotype may be due to the increasing accumulation of fluids in the body due to the lack of circulation.

The *sth* mutation was mapped at low resolution to a region on chromosome 8 [36], however none of the published markers in the region proved polymorphic for microsatellite analysis in our genetic backgrounds to allow finer resolution mapping. The more recent correlation of genetic and physical maps for zebrafish indicated that the minimal region contained a candidate locus, *smyd1b*, based on similarity to a morpholino knockdown phenotype [22] and the phenotype of a *smyd1b* mutation, *flatline* [20]. A complementation cross between heterozygous *still heart* and *flatline* adults produced mutant progeny, identifying *flatline* as allelic to *still heart* as a mutant at the *smyd1b* locus. Sequence analysis of *smyd1b* cDNA from *sth* mutant embryos revealed an insertion of 9 nucleotides that contain an in-frame stop codon between exon 1 and exon 2 (Fig 1H&1I). Genomic sequencing of *sth smyd1b* showed that the splice donor site of intron 1 has a transition mutation (Fig 1I&1J) that abolishes the function of this site and incorporates the stop codon that causes a premature truncation of the *Smyd1b* protein, effectively removing all of the functional domains (Fig 1K).

### SMYD1b is involved in the assembly of the sarcomeres in cardiac and fast-twitch muscle

While contraction of the heart in *sth* mutants is not seen, skeletal muscle is capable of some vibrational movement. One possibility for the reduced motility is that a lack of nutrient circulation due to a nonfunctional heart could disturb the development of normal skeletal muscle. The other possibility for the motility phenotype is that it is a direct effect of the *sth* mutation. To distinguish between these possibilities, we performed touch tests on young embryos (before the heart forms and begins beating at 24 hpf) derived from *sth* heterozygous parents and sorted them based on motility response. Of 548 embryos tested at 20 hpf, 156 (28%) were scored as deficient in touch response. After the sorted embryos were allowed to develop for several more hours, they were re-tested for the motility defect as well as for heartbeat. Of the embryos originally scored with a motility defect, 13% showed normal movement and heartbeat at the later time point, while none of the embryos scored as wild-type showed subsequent movement defect. Overall, 138 of the 548 (25%) embryos showed both motility and heart defects at the later time point, consistent with a single recessive locus with complete penetrance. Therefore, the vibrational movement and motility defect of *still heart* mutants is present before the heartbeat begins and is a defect of the *sth* mutation, not a secondary effect of a lack of circulation. In addition, it appears that development of the vascular system to the skeletal muscle is superficially unaffected in *sth* mutants (S1 Fig).

**Fig 2:**
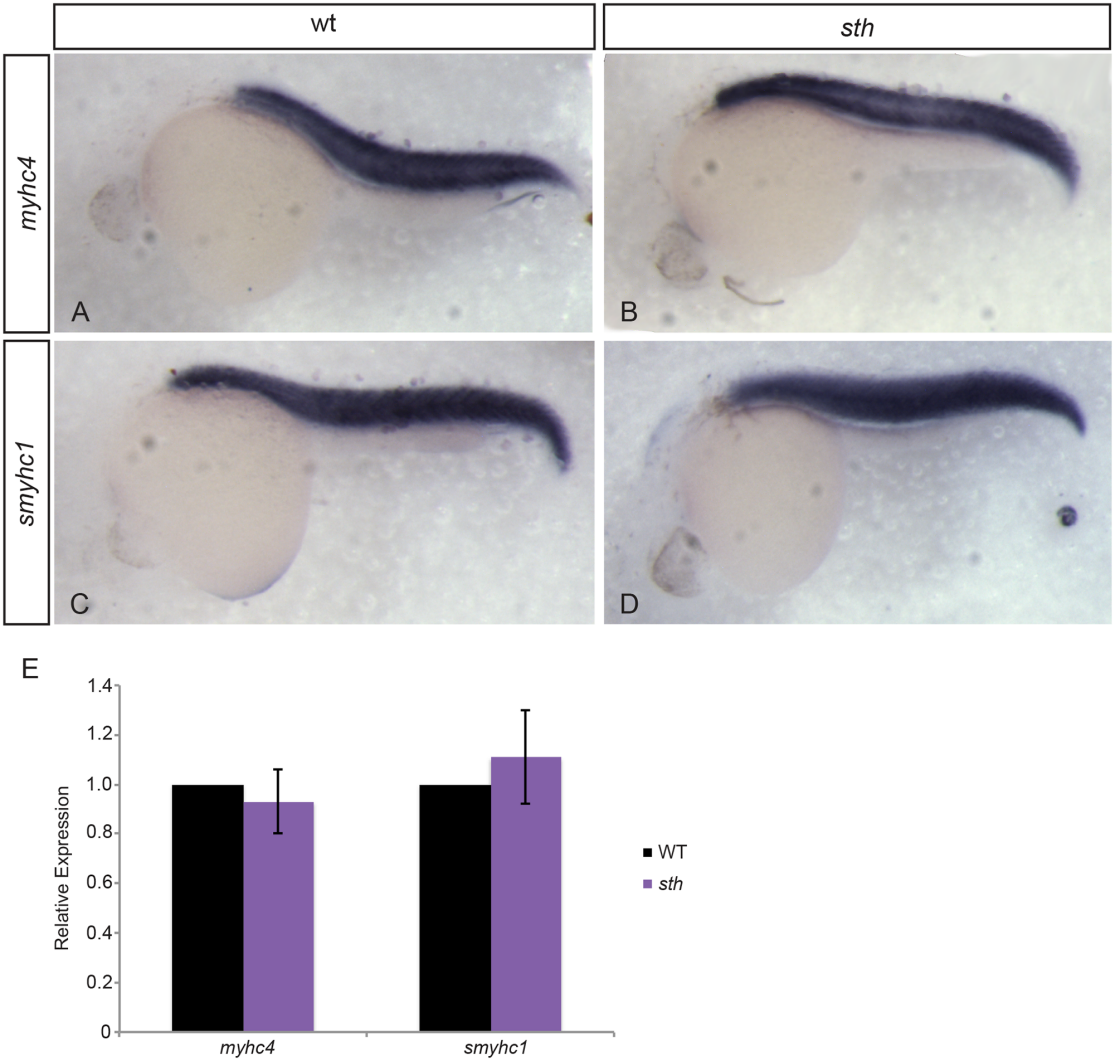
Myosin expression is unaffected by the absence of SMYD1b. At 24hpf, fast myosin (*myhc4*) expression in wild type (A) and *still heart* embryos (B) is similar within the trunk muscle. Slow myosin (*smyhc1*) expression in wild type (C) and *still heart* embryos (D) is also similar at 24hpf. Quantitative PCR on wild type and *sth* mutants for fast and slow myosin expression shows myosin expression is normal when compared to wild type embryos at 24hpf (E). (qPCR: n = 3, 30 embryos each time/phenotype. Error bars are standard deviation.).

The predicted protein structure of SMYD1b contains SET domains that have been associated with histone methyltransferase function [22, 23], however more recent work has shown that SMYD1b binds myosin and localizes to the M-line of sarcomeres after myocyte differentiation [20]. To determine whether SMYD1b is playing a role in muscle development as a HMT, responsible for myosin expression, or a myosin chaperone that is required for assembly and maintenance of the sarcomere, we examined myosin expression and incorporation into the sarcomere. *Still heart* mutants do not swim away after being touched, a normal evasion response to predators that is seen in their wild type siblings. The lack of an avoid response suggests that the *sth* mutation is affecting fast skeletal muscle fibers that are used for rapid swimming in response to an acute stimulus. We analyzed the expression levels of fast and slow-twitch myosin mRNA in *sth* mutants and wild type siblings and found that there were no significant differences in myosin expression between homozygous mutants and sibs (Fig 2A–2E).

**Fig 3:**
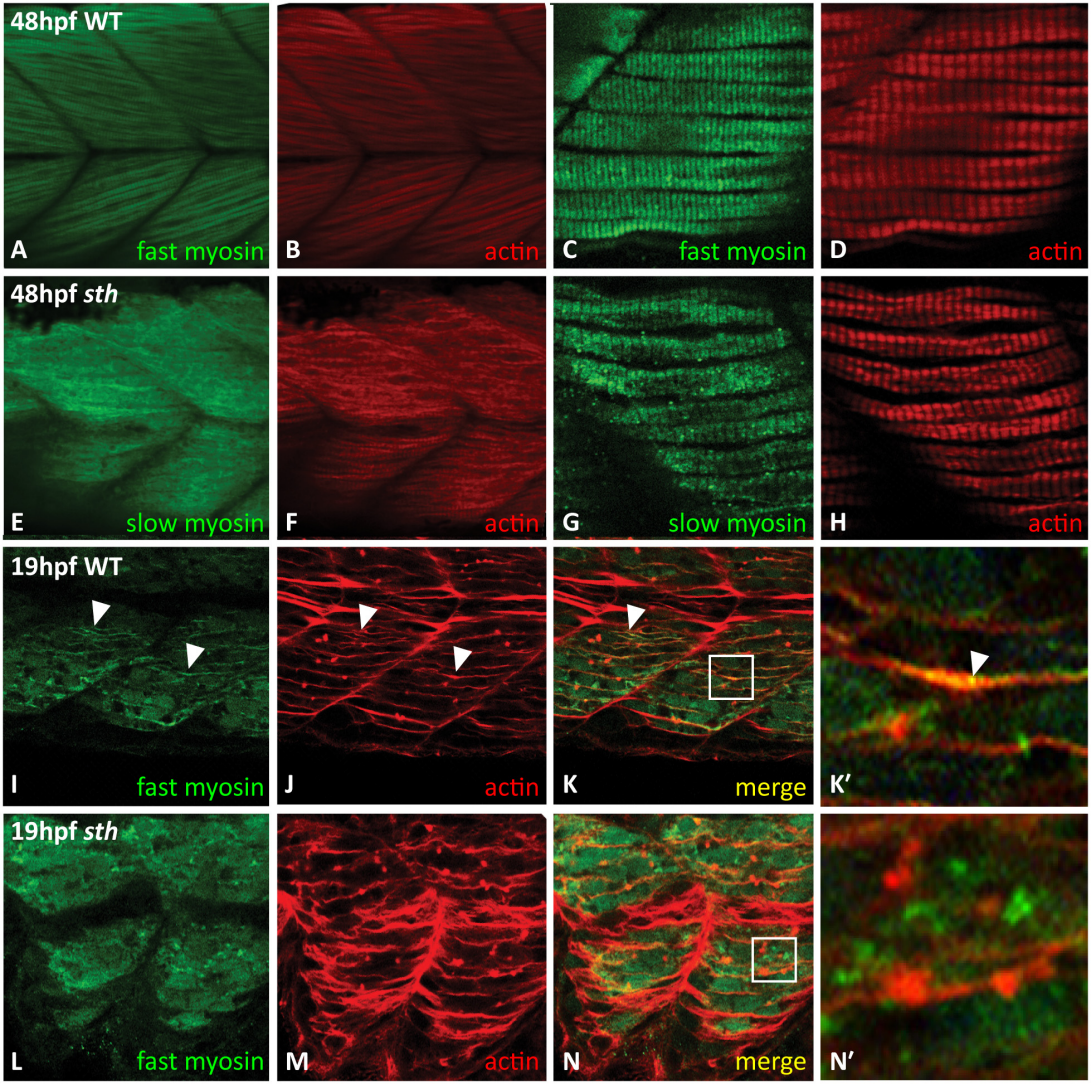
SMYD1b is required for fast myosin incorporation during sarcomere assembly. At 48hpf, fast myosin (F310—green) and actin (phalloidin—red) staining is visible in the premyofibrils in wt zebrafish tails (A&B), while absent from the premyofibrils in *sth* fast muscle tissue (C&D). Slow muscle (F59) develops normally in both wild type and *still heart* zebrafish at 48hpf (E&F, G&H). At 19hpf, in wild type embryos, fast myosin (F310) is beginning to be incorporated into the maturing myofibril (I) and overlaps (white arrowheads) with the developing actin (phalloidin) (J) fibers in trunk muscle (K, merge, K’ inset, white arrowhead). Fast myosin is not incorporated into the maturing premyofibril (L), although actin fibers are still present (M&N, N’ inset).

Comparison of fast and slow muscle fibers in wild type and *still heart* embryos shows that fast muscle fibers lack striations, indicative of sarcomeres, when compared to fast muscle in wild type siblings at 48 hpf (Fig 3A-3H). Slow muscle maintains striations throughout development although myofibrils eventually become disrupted as a secondary effect from the increasing disorganization surrounding fast muscle fibers (Fig 1G). Even though fast myosin is not incorporated into the sarcomere, fast myosin protein is still abundant in the muscle tissue [21]. This supports a model where SMYD1b is a chaperone-like protein that is involved in the assembly and maintenance of the sarcomere. To determine if SMYD1b is an assembly factor in sarcomere development, we examined early time points (15 and 19 hpf) in fast muscle sarcomere formation. Sarcomeres are absent in fast muscle in *sth* mutants at later stages [20], but it is not clear whether this is due to sarcomere breakdown in the absence of a maintenance role of SMYD1b, or if the sarcomeres never formed due to a lack of assembly by SMYD1b. If sarcomeres are present at the time when myosin is first incorporated, then SMYD1b is involved in the maintenance of sarcomeric proteins that later breakdown from stress of contraction and are never replaced. If myosin is not present at the beginning of sarcomere formation, then SMYD1b is an assembly factor that is required for the proper folding and incorporating of fast myosin into the sarcomere; if SMYD1b is an assembly factor, this does not rule out the possibility that it also has a role in maintenance of sarcomere proteins. At the 20 somite stage, the embryo contains both mature somites with formed sarcomeres and developing somites that have yet to build their sarcomeres. We examined fast myosin immunostaining at 14 and 20 somites in wild type and *still heart* embryos. At 14 somites, fast myosin was not detectable in any of the somites. However at 20 somites, premature actin/myosin fibers form between myosepta in wild type embryos (Fig 3I-3K). In *still heart* embryos, at 20 somites, actin fibers are present, but no fast myosin fibers can be detected in fast muscle tissue (Fig 3L-3N). The lack of fast myosin in the premature actin/myosin fiber during early sarcomere formation supports the model that SMYD1b is an assembly factor rather than a maintenance protein.

### SMYD1b works in tandem with myosin chaperones HSP90a1 and UNC45b to fold and incorporate fast myosin into the sarcomere

**Fig 4:**
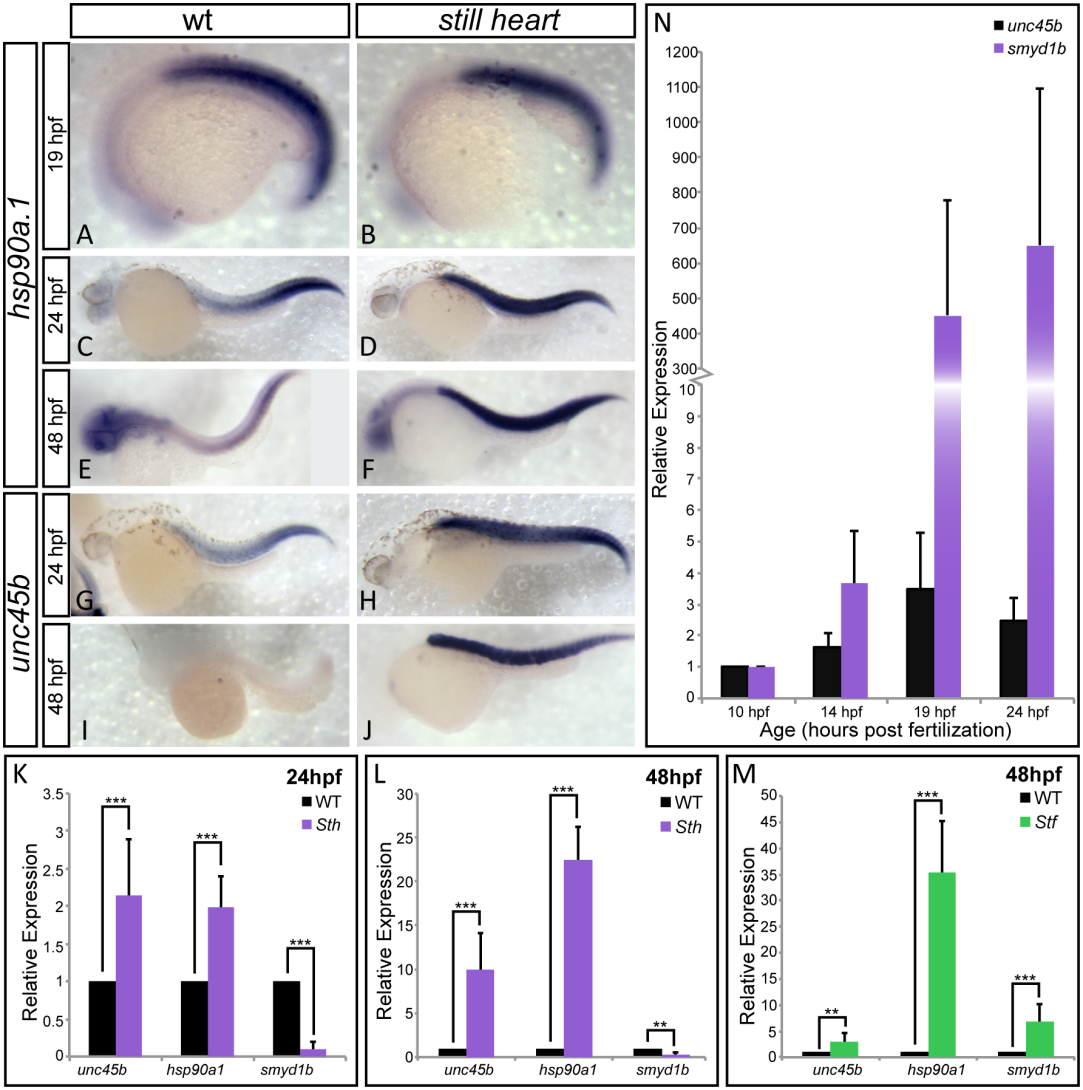
SMYD1b is co-regulated with other myosin chaperones HSP90a1 and UNC45b. In-situ hybridization staining of wild type and *still heart* embryos shows that *hsp90a1* expression is normal in *still heart* mutants, when compared to wild type embryos at 19hpf (A&B). However, the expression of *hsp90a1* and *unc45b* dramatically increases in *still heart* mutants at 24hpf (C-F & K) and 48hpf throughout the somites (G-J & M). Additionally, *smyd1b* expression increases significantly when UNC45b is absent in *steif* mutants (N), supporting co-regulation of these three genes. (O) A time course of *smyd1b* expression during muscle formation reveals that *smyd1b* is expressed early at 10hpf when *unc45b* is expressed and increases in its expression as muscle development progresses. Due to the rapid development of *unc45b* staining in the somites of the embryo in panel J, the head while present, has no background staining and is not clear in this focal plane. (qPCR: n = 3, 30 embryos each time/phenotype. Error bars are standard deviation.).

The absence of fast myosin incorporation suggests that SMYD1b is involved in incorporation of myosin into fast muscle sarcomeres. To test whether the *smyd1b* ^*sth*^ mutation is affecting sarcomere formation at the level of protein folding and assembly, and not transcriptional activation, we examined the mRNA expression levels of myosin chaperones *unc45b* and *hsp90a1*. These muscle chaperones will transcriptionally respond to the presence of misfolded client proteins but not the complete absence of sarcomeric structural proteins [20, 37]. *Still heart* mutants show a significant increase in *unc45b/hsp90a1* expression when compared to wild type siblings at 24 and 48 hpf (Fig 4C-4M). This increased expression is not due directly to the lack of SMYD1b, as at 19hpf the expression of *hsp90a1* resembles wild type expression (Fig 4A and 4B), suggesting that these chaperones are not co-regulated at the onset of fast myosin development. The increasing expression of these chaperones could be due the accumulation of misfolded myosins. We have observed the same trend of increasing chaperone expression of *smyd1b* and *hsp90a1* in *unc45b/steif* mutants (Fig 4N). Taken together, this supports a model where SMYD1b, UNC45b and HSP90a1 work together to properly fold and incorporate fast myosin into the sarcomere and exhibit a transcriptional up-regulation when myosin is not incorporated/folded in mutant myofibers.

Although the lack of SMYD1b predominantly shows defects in fast myosin incorporation, we analyzed the expression of *smyd1b* in comparison to *unc45b* in wild type embryos. *Smyd1b* is first detectable by *in situ hybridization* at 10hpf, at the time when *unc45b* is expressed and working with non-muscle myosin to build the premature sarcomere [16]. The expression of *smyd1b* 9 hours before fast muscle begins to develop (Fig 4O), suggests that SMYD1b may have an earlier muscle-myosin independent function during muscle development but such an early role remains unclear.

## Discussion

The sarcomere is a complex structure that requires the coordination and organization of hundreds of proteins for assembly and maintenance of this contractile unit. The discovery of muscle factors that have a role in both cardiac and skeletal sarcomere formation, suggests both muscle types have early development components in common, but later diverge and specialize during development. However, the precise molecular role of the majority of the factors in the formation and maintenance of the sarcomere remain unknown [14, 15]. Here we demonstrate that the histone methyltransferase, SMYD1b, is a chaperone-like assembly protein that is crucial to the assembly of the sarcomere with regards to fast but not slow muscle myosin in striated muscle.

It has been clear over the last few years that the Smyd family of proteins plays a role in muscle development but their molecular role and potential cofactors are relatively unknown [11, 25, 38]. SMYD2 is indirectly responsible for TITIN stability in the sarcomere by methylating HSP90a1 and forming a complex with the two during sarcomere formation [11]. We show that SMYD1b does not affect myosin mRNA expression, as would be expected from a chromatin modifying protein, but instead has a role in fast myosin incorporation into the sarcomere. It has been suggested that SMYD1b has a chaperone-like function and may be acting as an assembly or maintenance protein [20, 21]. This speculation generates three models for SMYD1b function. The first is that SMYD1b is involved early for establishment of the sarcomere, the second is that it is involved later for maintenance and the third model requires SMYD1b for both assembly and maintenance. To determine which model is most likely, we examined the earliest stages of myosin incorporation as an indirect measure of SMYD1b necessity. Our results show that fast, but not slow, muscle myosin is not incorporated into the sarcomere in the absence of SMYD1b, and it is at least an assembly protein specific to fast muscle tissue.

In both *flatline* and *still heart*, an increased expression in HSP90 and UNC45b is seen where SMYD1b is absent [20]. Although myosin chaperones HSP90, UNC45b and SMYD1b show evidence of co-regulation and have been suggested to work together to fold and incorporate myosin, little is known about whether SMYD1b interacts directly with the others, or functions independently at the same stage of assembly [20, 21]. Two models may explain how these three chaperones work together during sarcomere development. The first model is that HSP90, UNC45b and SMYD1b all physically interact and work together to fold and incorporate fast myosin into the sarcomere. In this case, we hypothesize that the MYND domain of SMYD1b is important for binding other proteins such as UNC45b or HSP90a1. The second model is that HSP90a1 and UNC45b interact with SMYD1b indirectly through contacts with fast myosin, and that myosin is passed on in an assembly line type fashion. We can conclude based on the increased expression of *hsp90a1*, *unc45b* or *smyd1b* in the absence of either *smyd1b* or *unc45b*, that these three chaperones are required for fast myosin folding and incorporation. The increased expression of these chaperones when myosin is not incorporation is an unfolded protein response that would eventually lead to a negative feedback loop on myosin if myosin cannot be properly folded. We see evidence of this regulation when either of these chaperones is missing; there is a decrease in the target myosin protein levels when the expression of the myosin chaperones is elevated [20]. Overexpression of *unc45b* leads to similar thick filament defects with decreased levels of myosin protein in the developing myofibers, however the mechanism behind how abundant UNC45b levels disrupts the myofibers is unknown [7, 39].

It is interesting to note that *smyd1b* is expressed hours before fast muscle begins to develop, but at the same time point where *unc45b* is expressed and has been suggested to be involved in assembly of the non-muscle myosin (NMM) scaffold of the premature sarcomere. It is unclear what SMYD1b’s function may be early in development, but perhaps SMYD1b aids UNC45b in folding and setting up the organization of NMM in the early sarcomere. The dramatic increase in *smyd1b* at 19hpf could be due to the onset of developing fast muscle where SMYD1b is required for sarcomere assembly while *unc45b* functions in both fast and slow muscle.

We have demonstrated the SMYD1b is co-regulated with myosin chaperones UNC45b and HSP90a1 and that all three are required for fast muscle development, specifically SMYD1b is responsible for sarcomere assembly in fast myofiber formation. Additionally, we have shown that SMYD1b has other roles that are myosin independent such as controlling heart morphogenesis. It is still not clear what SMYD1b is doing very early in muscle development before fast muscle begins to form, but it may have a shared role with UNC45b in NMM patterning of the sarcomere. Understanding how SMYD1b interacts with the other myosin chaperones and the order of their function, may reveal a coordinated chaperone system with SMYD proteins having early roles that stimulate the function of other chaperone proteins in muscle development.

## Supporting Information

S1 Fig Vasculature is normal in *still heart* mutants at 48hpf. Lateral view of 48hpf zebrafish vasculature demonstrates a repeating network of arteries and veins that highlight each somite in the trunk. Comparing vasculature organization between wild type (A) and *still heart* (B), there are no visible differences. (n = 10 embryos)
(TIF)

S1 Table Primer Sequences used for analysis of SMYD1b’s role in muscle development. (PDF)
