# Correction: *Still Heart* Encodes a Structural HMT, SMYD1b, with Chaperone-Like Function during Fast Muscle Sarcomere Assembly

**DOI:** 10.1371/journal.pone.0148027

**Published:** 2016-01-22

**Authors:** Kendal Prill, Pamela Windsor Reid, Serene L. Wohlgemuth, David B. Pilgrim

In Fig 3, the images for panels C&D and E&F are switched. Please see the corrected Fig 3 here.

**Fig 3:**
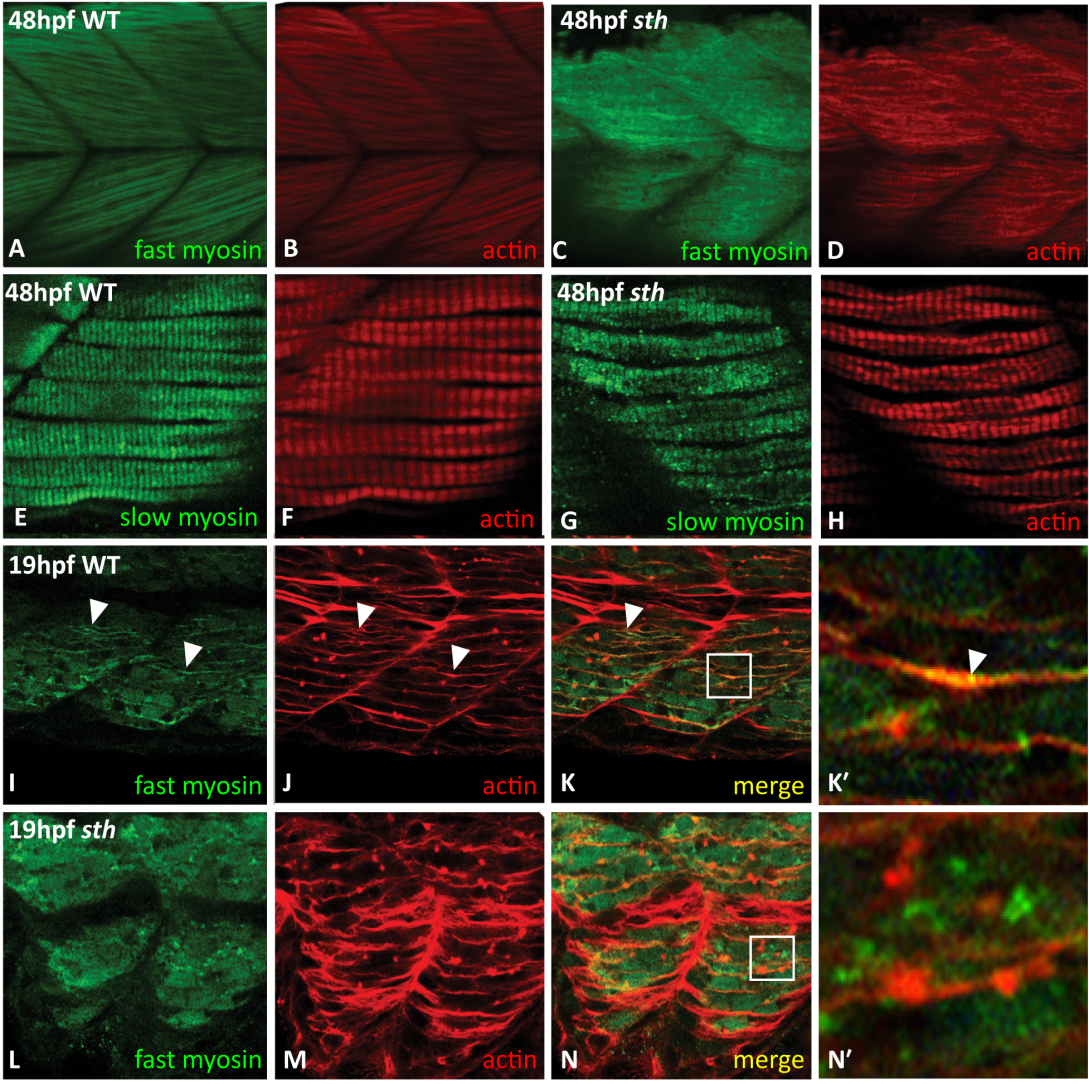
SMYD1b is required for fast myosin incorporation during sarcomere assembly. At 48hpf, fast myosin (F310—green) and actin (phalloidin—red) staining is visible in the premyofibrils in wt zebrafish tails (A&B), while absent from the premyofibrils in sth fast muscle tissue (C&D). Slow muscle (F59) develops normally in both wild type and still heart zebrafish at 48hpf (E&F, G&H). At 19hpf, in wild type embryos, fast myosin (F310) is beginning to be incorporated into the maturing myofibril (I) and overlaps (white arrowheads) with the developing actin (phalloidin) (J) fibers in trunk muscle (K, merge, K’ inset, white arrowhead). Fast myosin is not incorporated into the maturing premyofibril (L), although actin fibers are still present (M&N, N’ inset).
