# Supplementary figures and images for: Still Heart Encodes a Structural HMT, SMYD1b, with Chaperone-Like Function during Fast Muscle Sarcomere Assembly
Source: PLoS One. 2015 Nov 6;10(11):e0142528. doi: 10.1371/journal.pone.0142528 (PMC4636364; doi:10.1371/journal.pone.0142528)

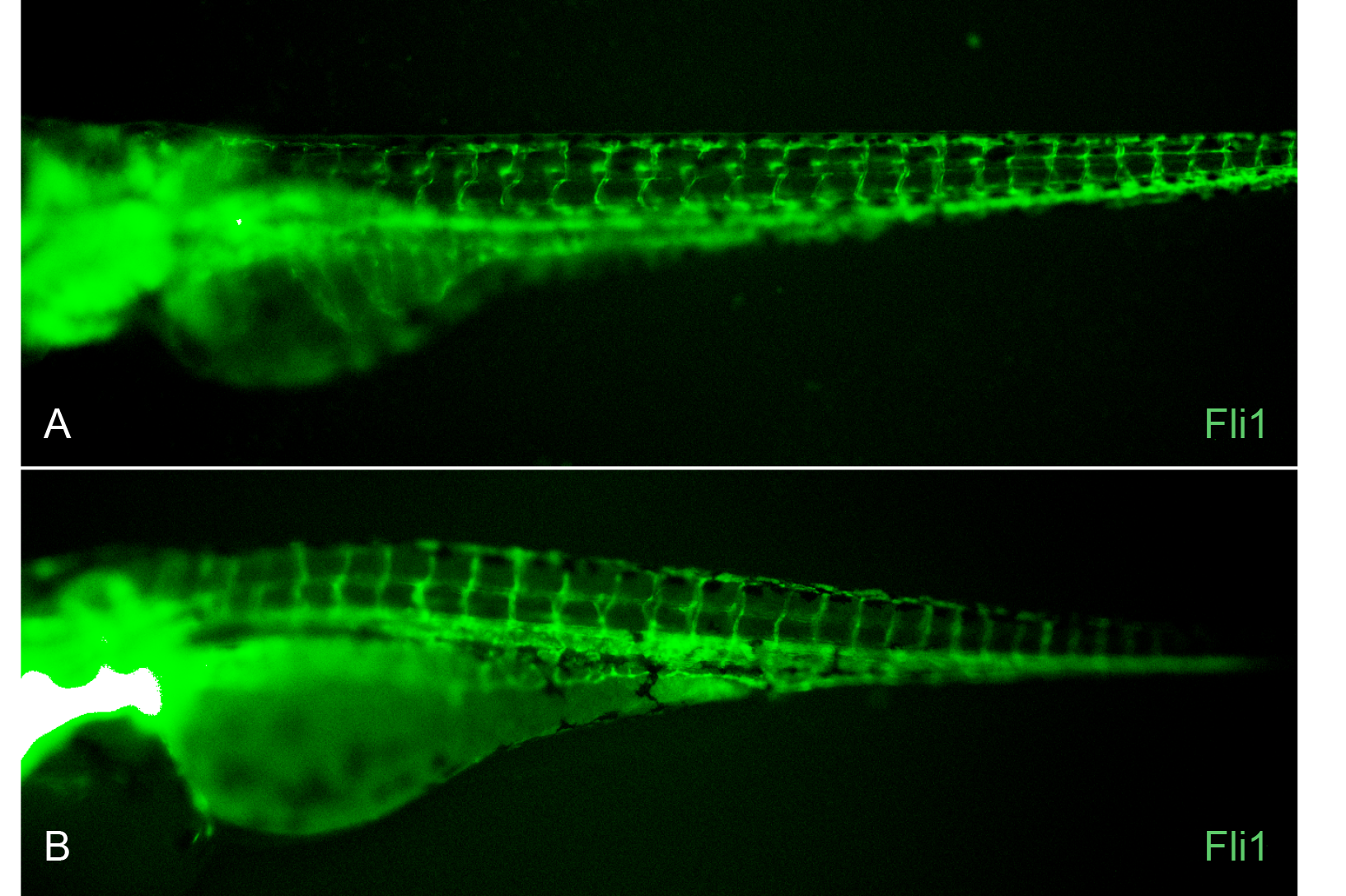

Supplement: S1 Fig — Lateral view of 48hpf zebrafish vasculature demonstrates a repeating network of arteries and veins that highlight each somite in the trunk. Comparing vasculature organization between wild type (A) and still heart (B), there are no visible differences. (n = 10 embryos) (TIF) [file pone.0142528.s001.tif]
