# Supplementary material for: Still Heart Encodes a Structural HMT, SMYD1b, with Chaperone-Like Function during Fast Muscle Sarcomere Assembly
Source: PLoS One. 2015 Nov 6;10(11):e0142528. doi: 10.1371/journal.pone.0142528 (PMC4636364; doi:10.1371/journal.pone.0142528)
Supplement: S1 Table — (PDF) [file pone.0142528.s002.pdf]

S1 Table 1. Primer sequences used for analysis of SMYD1b's role in muscle development.

Primer	Sequence 5' → 3'	Purpose
gata5 FWD	CTA CCG GGA AGG AGG TCC AGT ATA G	ISH
gata5 REV T7	TAA TAC GAC TCA CTA TAG GGG CCA CCA TAA ATC AAG GAG GAA AAG	ISH
gata4 FWD	CAT AAC TCG ACT TCT CCG GTG TAC G	ISH
gata4 REV T3	CAT TAA CCC TCA CTA AAG GGA AGT TCC ACA CTT CAC TCT TGG AGC TG	ISH
Hsp90a.1 FWD	TCT TTT GCG CTA CTA CAC TTC AGC TTC	ISH
HSP90a.1 REV T7	TAA TAC GAC TCA CTA TAG GGA TAA AAT GCA AGA GCA GAC ACA CAA GG	ISH
Myh4 FWD	TCT GAA GAA GCT GAG GAA CAA G	qPCR
Myh4 REV	TTG ACC TGG GAC TCA GAA ATG	qPCR
Smyhc1 FWD	CCA GAC TGA AGA AGA CCG TAA G	qPCR
Smyhc1 REV	GGA ACT TGC CCA GGT TAG AA	qPCR
Unc45b FWD	ACC TCC TTG CAG CAA ACT	qPCR
Unc45b REV	AGG ATC ATG AAC AGA TCA GAC AA	qPCR
Hsp90a1 FWD	GGA TGA GCT GAA GGC CAA ATA	qPCR
Hsp90a1 REV	GTA TGT GCT GGT GAC GAT ACA G	qPCR
Smyd1b FWD	ATG GAG AAG GCC AGG ATA GA	qPCR
Smyd1b REV	GGT ACA ACA CAC ACG CAG ATA	qPCR
Ef1a FWD	CCT TCG TCC CAA TTT CAG G	qPCR
Ef1a REV	CCT TGA ACC AGC CCA TGT	qPCR
Smyd1b Exon 1 FWD	GGT ACA GCT GAA GAC TGG TCT ATT TGA	Sequencing
Smyd1b Exon 1 REV	TAT CCA GCA CCC TTC ACC TGT AAT CAT	Sequencing
Smyd1b Exon 2 REV	CAG GTT TGC GCA GTA CTG TGA CAA	Sequencing

ISH, in-situ hybridization; qPCR, quantitative polymerase chain reaction
